# Human leukocyte antigen-DQ risk heterodimeric haplotypes of left ventricular dysfunction in cardiac sarcoidosis: an autoimmune view of its role

**DOI:** 10.1038/s41598-023-46915-1

**Published:** 2023-11-13

**Authors:** Hironori Yamamoto, Yohei Miyashita, Hitoshi Minamiguchi, Kazuyoshi Hosomichi, Shohei Yoshida, Hidetaka Kioka, Haruki Shinomiya, Haruno Nagata, Kenji Onoue, Masato Kawasaki, Yuki Kuramoto, Akihiro Nomura, Yuichiro Toma, Tetsuya Watanabe, Takahisa Yamada, Yasuki Ishihara, Miho Nagata, Hisakazu Kato, Hideyuki Hakui, Yoshihiko Saito, Yoshihiro Asano, Yasushi Sakata

**Affiliations:** 1 Department of Cardiovascular Medicine, Osaka University Graduate School of Medicine, 2-2 Yamadaoka, Suita, Osaka 565-0871 Japan; 2 Department of Legal Medicine, Osaka University Graduate School of Medicine, Suita, Osaka 565-0871 Japan; 3 Laboratory of Computational Genomics, School of Life Science, Tokyo University of Pharmacy and Life Sciences, Hachioji, Tokyo 192-0392 Japan; 4 Department of Cardiovascular Medicine, Kanazawa University Hospital, Kanazawa, Ishikawa 920-8641 Japan; 5 Department of Cardiovascular Medicine, University of the Ryukyus Graduate School of Medicine, Nakagami, Okinawa 903-0215 Japan; 6 Department of Cardiovascular Medicine, Nara Medical University, Kashihara, Nara 634-8522 Japan; 7 Department of Cardiology, Osaka General Medical Center, Osaka, Osaka 558-8558 Japan; 8 Innovative Research Center, Kanazawa University School of Medicine, Kanazawa, Ishikawa 920-8641 Japan; 9 The 1st Department of Oral and Maxillofacial Surgery, Osaka University Graduate School of Medicine, Suita, Osaka 565-0871 Japan; 10 Department of Medical Ethics and Medical Genetics, Kyoto University Graduate School of Medicine, Kyoto, Kyoto 606-8501 Japan; 11 Department of Medical Biochemistry, Osaka University Graduate School of Medicine, Suita, Osaka 565-0871 Japan; 12 Department of Cardiovascular Medicine, Nara Prefecture Seiwa Medical Center, Nara, Nara 636-0802 Japan; 13 Department of Genomic Medicine, National Cerebral and Cardiovascular Center, Suita, Osaka 564-8565 Japan

**Keywords:** Computational biology and bioinformatics, Immunology, Cardiology

## Abstract

Cardiac sarcoidosis (CS) is the scarring of heart muscles by autoimmunity, leading to heart abnormalities and patients with sarcoidosis with cardiac involvements have poor prognoses. Due to the small number of patients, it is difficult to stratify all patients of CS by human leukocyte antigen (HLA) analysis. We focused on the structure of antigen-recognizing pockets in heterodimeric HLA-class II, in addition to DNA sequences, and extracted high-affinity combinations of antigenic epitopes from candidate autoantigen proteins and HLA. Four HLA heterodimer-haplotypes (DQA1*05:03/05:05/05:06/05:08-DQB1*03:01) were identified in 10 of 68 cases. Nine of the 10 patients had low left ventricular ejection fraction (< 50%). Fourteen amino-acid sequences constituting four HLA anchor pockets encoded by the HLA haplotypes were all common, suggesting DQA1*05:0X-DQB1*03:01 exhibit one group of heterodimeric haplotypes. The heterodimeric haplotypes recognized eight epitopes from different proteins. Assuming that autoimmune mechanisms might be activated by molecular mimicry, we searched for bacterial species having peptide sequences homologous to the eight epitopes. Within the peptide epitopes form the SLC25A4 and DSG2, high-homology sequences were found in *Cutibacterium acnes* and *Mycobacterium tuberculosis*, respectively. In this study, we detected the risk heterodimeric haplotypes of ventricular dysfunction in CS by searching for high-affinity HLA-class II and antigenic epitopes from candidate cardiac proteins.

## Introduction

Sarcoidosis is a systemic granulomatous autoimmune disease affecting various organs, including the heart^1^. Cardiac sarcoidosis (CS), or sarcoidosis with cardiac phenotypes, exhibits three major phenotypes in the heart, namely, complete atrioventricular block (CAVB), ventricular arrhythmia (VT/VF), and left ventricular systolic dysfunction (low left ventricular ejection fraction; LVEF). These cardiac phenotypes indicate poor prognosis for patients with sarcoidosis^2^. Although Human Leukocyte Antigen class II (HLA-II) genes have been reported to be associated with sarcoidosis incidence^3–6^, the pathological mechanisms and risk factors associated with these cardiac phenotypes have not been elucidated.

HLA-II molecules can present self-antigens to CD4-positive T-cells, and their mechanism in autoimmune reactions is partially known. Autoimmune reactions can be triggered by peptides of foreign pathogens that mimic self-peptides, and susceptibility to autoimmune diseases is often associated with specific HLA-II alleles that are associated with self-antigen presentation to auto-reactive T-cells^7^. Self-antigen recognition could be acquired via molecular mimicry, which occurs when similarities between foreign and autologous antigens favour activation of auto-reactive T-cells in susceptible individuals^8^. T-cell activation via molecular mimicry is mediated by HLA-II molecules whose binding affinity to foreign and autologous antigenic epitopes could affect antigen presentation, immunogenicity, and cross-reactivity^9,10^. Some autoimmune diseases, such as systemic scleroderma^11^, Guillain–Barré syndrome^12^, and autoimmune retinitis^13^ have been reported to be triggered by molecular mimicry, where infection with certain foreign pathogens was suggested to be the trigger. Moreover, these diseases often develop in adulthood and have been reported to be associated with specific HLA alleles, which are also common in CS. Given these, environmental factors, especially exposure to foreign pathogens, may be involved in the cardiac phenotype.

In this study, we considered two strategies to identify HLAs that could be risk factors for the development of CS phenotypes. The first strategy was to identify HLAs that were significantly enriched in patients with CS and statistically correlated with the CS phenotypes. The second strategy was to identify HLAs with high auto-immunogenicity using the binding affinity of HLA molecules to autologous cardiac proteins and to search for phenotypic characteristics of cases with these HLAs. Therefore, we identified probable CS-associated autoantigens using a bioinformatics tool that employs tailored machine learning strategies to integrate predictors trained on binding affinity data and mass spectrometry experiments^14^. Furthermore, we demonstrated that some of the HLA-DQ heterodimer-haplotypes could be grouped into an HLA-DQ heterodimeric haplotypes based on the binding affinity profile. Subsequently, the association between the HLA-DQ heterodimeric haplotypes and cardiac phenotypes in patients with CS was examined. Finally, we comprehensively analysed foreign antigens that were highly homologous to the identified autoantigens associated with the disease.

## Results

### Detection of risk HLA alleles associated with CS susceptibility or CS phenotypes

As the first strategy, we performed 4-digit HLA typing by target resequencing of 11 classical HLA genes in 68 patients with CS and 311 controls. In the patient group, we detected 14 HLA-A, 28 HLA-B, 16 HLA-C, 4 HLA-DPA1, 13 HLA-DPB1, 13 HLA-DQA1, 11 HLA-DQB1, 18 HLA-DRB1, 4 HLA-DRB3, 2 HLA-DRB4, and 2 HLA-DRB5 alleles. We then conducted association analysis of these alleles with disease incidence.

**Figure 1:**
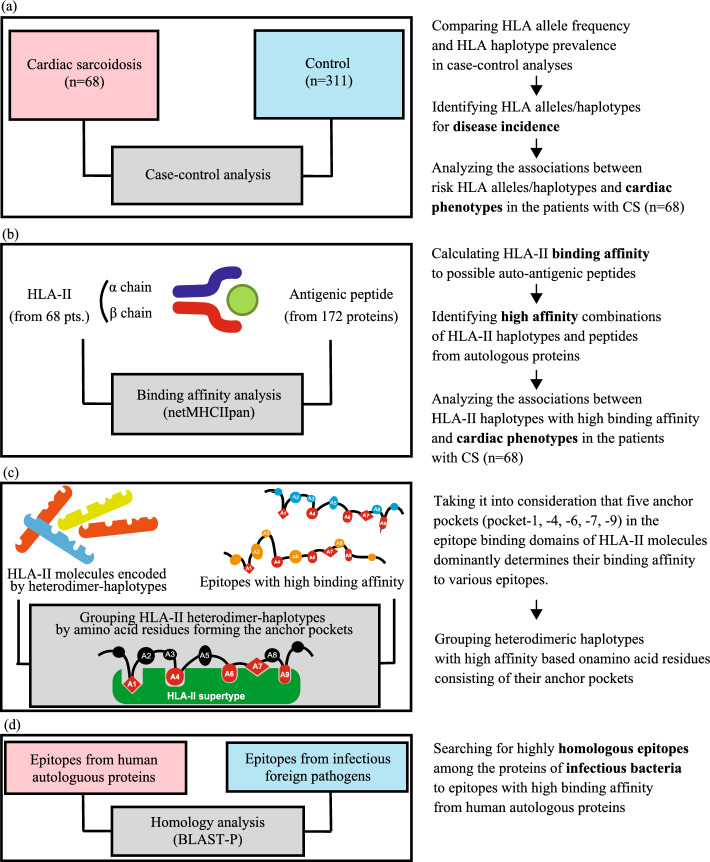
Research strategies for identifying risk HLA of cardiac phenotypes in patients with CS. (**a**) Following conventional HLA analyses, prevalence of HLA alleles or haplotypes were compared between case and control groups. Associations between the risk alleles or haplotypes detected and cardiac phenotypes. (**b**) From an autoimmune perspective, the hetero-dimeric structure of HLA-II molecules (of the 68 patients with CS) and their binding affinity (BA) to possible antigenic epitopes (of 172 proteins) were taken into consideration. After the comprehensive calculation of BA, the combinations of HLA-II haplotypes and epitopes with high BA were extracted. Associations between HLA-II haplotypes with high BA and cardiac phenotypes. (**c**) Five anchor pockets in the epitope-binding domains of HLA-II molecules determine their BA to various epitopes. HLA-II haplotypes with similar BA patterns were grouped into an HLA heterodimeric haplotypes. This process was validated by homology of their amino acid residues in the peptide binding domains. (**d**) The possibility of auto-immunogenicity by the risk HLA haplotypes (or heterodimeric haplotypes) was considered with the mechanism of molecular mimicry. We used BLAST-P to look for epitopes from proteins expressed in infectious bacteria, which were homologous to the human epitopes with high BA.

Given that HLA-II alleles have been shown to be associated with sarcoidosis incidence, we sought to identify HLA alleles for each locus that were significantly enriched in the CS group compared to that in the control group. These analyses were performed as reported in previous HLA studies of autoimmune diseases^11,15,16^. (Fig. 1a). In this study, the prevalence of the DQB1*06:01 allele, a previously reported risk HLA allele for CS incidence^17^, was significantly higher in the HLA-DQB1 locus in the group of patients with CS (56%) than that in the control group (33%) (Table 1). Similarly, prevalence of the DRB1*08:03 (CS vs. control: 43% vs. 16%) (Supplemental Table S1) and DQA1*01:03 alleles (CS vs. healthy control: 59% vs. 38%) (Supplemental Table S2) were significantly higher in the CS group than in the control group. Additionally, we detected the association of HLA class I genes with CS incidence. In the HLA-A locus, the prevalence of HLA-A*11:01 allele was significantly higher in the CS group (26%) than that in the control group (12%) (Supplemental Table S3). In the HLA-C locus, the prevalence of HLA-C*03:04 allele was significantly lower in the CS group (7%) than that in the control group (25%) (Supplemental Table S4). Thus, the HLA-C*03:04 allele may be a protective factor of CS incidence, while HLA-DQB1*06:01, -DRB1*08:03, -DQA1*01:03, and -A*11:01 alleles were risk factors. Similar single allele-based analyses for the other HLA-II loci (HLA-DPA1, -DPB1, -DRB3, -DRB4, or -DRB5) and the HLA-B locus did not reveal any significant risk alleles (Supplemental Table S5).

**Table 1:** Association analysis for the risk HLA-*DQB1* allele of CS incidence.

Allele	Cardiac sarcoidosis (n = 68)	Control (n = 311)	P_adj
DQB1*06:01	38 (56%)	105 (34%)	0.096*
DQB1*05:01	1 (1.5%)	33 (11%)	n.s
DQB1*04:01	21 (31%)	68 (22%)	n.s
DQB1*05:03	1 (1.5%)	17 (5.5%)	n.s
DQB1*04:02	8 (12%)	25 (8.0%)	n.s
DQB1*06:02	7 (10%)	46 (15%)	n.s
DQB1*03:02	11 (16%)	57 (18%)	n.s
DQB1*06:03	2 (2.9%)	14 (4.5%)	n.s
DQB1*03:03	20 (29%)	100 (32%)	n.s
DQB1*03:01	15 (22%)	69 (22%)	n.s
DQB1*06:04	5 (7.4%)	26 (8.4%)	n.s

*n.s.* no significant difference.

*Significance after the correction for multiple testing (Bonferroni correction); P_adj. < 0.05.

Subsequently, we attempted to detect HLA alleles associated with cardiac phenotypes in patients with CS. We focused on the five HLA alleles (DQB1*06:01, DRB1*08:03, DQA1*01:03, A*11:01, and C*03:04) that were found to be risk or protective HLA alleles for CS incidence. A total of 68 patients with CS were divided into two groups according to the presence of each allele. The differences in clinical characteristics and the prevalence of clinical phenotypes were compared between the two groups. There was no considerable association between the presence of DQB1*06:01 allele and the prevalence of any cardiac phenotypes in patients with CS (Table 2). Similarly, there were no considerable associations between either DRB1*08:03 (Supplemental Table S6), DQA1*01:03 (Supplemental Table S7), A*11:01 (Supplemental Table S8), or C*03:04 (Supplemental Table S9) alleles and any cardiac phenotypes in patients with CS. Hence, these HLA alleles were associated with CS susceptibility, but they could not be used to stratify cardiac phenotypes in patients with CS.

**Table 2:** Association analysis between the risk HLA allele of DQB1*06:01 and clinical phenotypes.

	With DQB1*06:01 (n = 38)	Without DQB1*06:01 (n = 30)	P
Clinical characteristics			
Age of onset (years)	64 [56–72]	60 [52–69]	0.42
Gender (female)	23 (61%)	20 (67%)	0.62
Clinical phenotypes			
CAVB	18 (47%)	12 (40%)	0.63
VT or VF	16 (42%)	9 (30%)	0.33
Sustained VT or VF	10 (26%)	7 (23%)	1.00
LVEF (%)	45 [33–57]	46 [35–63]	0.60
Low LVEF	24 (63%)	17 (57%)	0.63
Cardiac device implantation	29 (76%)	20 (67%)	0.42
Successful corticosteroid therapy	15 (44%)	6 (27%)	0.26

*CAVB* complete atrioventricular block, *VT* ventricular tachycardia, *VF* ventricular fibrillation, *LVEF* left ventricular ejection fraction, *Low LVEF* the patients with LVEF < 50% in the echocardiography at the enrolment.

### Detection of risk HLA heterodimer-haplotypes associated with CS susceptibility or cardiac phenotypes of CS

HLA-II alleles are risk factors associated with sarcoidosis incidence, based on single allele analyses^3–6^. However, analyses based on HLA heterodimer-haplotypes might be reasonable considering that the epitope-binding domain of HLA-II molecules has a heterodimeric structure comprising an α chain (encoded by the *A*-genes; e.g., *HLA-DPA1* or *-DQA1* genes) and a β chain (encoded by the *B*-genes; e.g., *HLA-DPB1* or -*DQB1* genes). We therefore created a list of possible combinations of the *A* and *B* gene alleles as “heterodimer-haplotypes” (e.g., *HLA-DPA1/DPB1 or -DQA1/DQB1)*. A total of 31 HLA-DP and 67 HLA-DQ heterodimer-haplotypes were identified in the CS group. We conducted association analyses of these heterodimer-haplotypes with CS incidence and cardiac phenotypes (Fig. 1a). The prevalence of DQA1*03:03/DQB1*06:01, DQA1*01:03/DQB1*04:01, and DPA1*02:02/DPB1*09:01 heterodimer-haplotypes were significantly higher in the CS group than in the control group (Tables 3, 4). Subsequently, we studied the effects of the presence of DQA1*03:03/DQB1*06:01 (Table 5), DQA1*01:03/DQB1*04:01 (Supplemental Table S10), and DPA1*02:02/DPB1*09:01 (Supplemental Table S11) haplotypes on the prevalence of cardiac phenotypes in patients with CS. However, we did not detect any significant associations.

**Table 3:** Association analysis for the risk HLA-*DQ* haplotypes of CS incidence.

Haplotype	Cardiac sarcoidosis (n = 68)	Control (n = 311)	P_adj
DQA1*03:03/DQB1*06:01	16 (23%)	13 (4.2%)	1.5E-04 *
DQA1*01:03/DQB1*04:01	12 (17%)	9 (2.9%)	2.3E-03 *
DQA1*01:03/DQB1*06:01	38 (55%)	105 (34%)	n.s
DQA1*01:01/DQB1*05:01	1 (1.5%)	28 (9.0%)	n.s
DQA1*03:02/DQB1*06:01	10 (14%)	21 (6.8%)	n.s
DQA1*05:05/DQB1*06:02	2 (2.9%)	1 (0.3%)	n.s
DQA1*01:03/DQB1*03:03	10 (14%)	24 (7.7%)	n.s
DQA1*03:03/DQB1*03:02	4 (5.9%)	7 (2.3%)	n.s
DQA1*03:03/DQB1*04:01	21 (31%)	68 (22%)	n.s
DQA1*01:03/DQB1*03:01	5 (7.4%)	11 (3.5%)	n.s

A total of 67 HLA-DQ haplotypes detected in the group with patients suffering from CS, ordered by P values; the results of the top 10 haplotypes are shown.

*n.s.* no significant difference.

*Significance after the correction for multiple testing (Bonferroni correction); P_adj. < 0.05.

**Table 4:** Association analysis for the risk HLA-DP haplotypes of CS incidence.

Haplotype	Cardiac sarcoidosis (n = 68)	Control (n = 311)	P_adj
DPA1*02:02/DPB1*09:01	12 (18%)	16 (5.1%)	0.038 *
DPA1*02:02/DPB1*02:02	7 (10%)	8 (2.6%)	n.s
DPA1*02:01/DPB1*02:01	1 (1.5%)	34 (11%)	n.s
DPA1*02:01/DPB1*05:01	17 (25%)	41 (13%)	n.s
DPA1*02:02/DPB1*06:01	2 (7%)	0 (0.0%)	n.s
DPA1*02:01/DPB1*02:02	3 (4.4%)	2 (0.6%)	n.s
DPA1*01:03/DPB1*06:01	2 (2.9%)	1 (0.3%)	n.s
DPA1*02:02/DPB1*05:01	48 (71%)	184 (59%)	n.s
DPA1*02:02/DPB1*13:01	2 (2.9%)	2 (0.6%)	n.s
DPA1*01:03/DPB1*19:01	1 (1.5%)	0 (0.0%)	n.s

A total of 31 HLA-DP haplotypes detected in the group with patients suffering from CS, ordered by P values; the results of the top 10 haplotypes are shown.

*n.s.* no significant difference.

*Significance after the correction for multiple testing (Bonferroni correction); P_adj. < 0.05.

**Table 5:** Association analysis between the risk HLA haplotype, DQA1*03:03/DQB1*06:01 and clinical phenotypes.

	With DQA1*03:03/DQB1*06:01 (n = 16)	Without DQA1*03:03/DQB1*06:01 (n = 52)	P
Clinical characteristics			
Age of onset (years)	66 [56–75]	62 [53–70]	0.51
Gender (female)	10 (63%)	33 (63%)	1.00
Clinical phenotypes			
CAVB	10 (63%)	20 (38%)	0.15
VT or VF	9 (56%)	16 (31%)	0.08
Sustained VT or VF	5 (31%)	12 (23%)	0.52
LVEF (%)	44 [34–56]	46 [34–60]	0.61
Low LVEF	11 (69%)	30 (58%)	0.56
Cardiac device implantation	12 (75%)	37 (71%)	1.00
Successful corticosteroid therapy	8 (57%)	14 (33%)	0.13

*CAVB* complete atrioventricular block, *VT* ventricular tachycardia, *VF* ventricular fibrillation, *LVEF* left ventricular ejection fraction, *Low LVEF* the patients with LVEF < 50% in the echocardiography at the enrollment.

In summary, the HLA single alleles and heterodimer-haplotypes that we identified correlated with CS susceptibility. Nonetheless, these HLAs did not correlate significantly with the cardiac phenotypes of CS. We must thus explore the risk HLAs of CS phenotypes.

### Calculation and processing of the binding affinity between HLA molecules and autologous peptide sequences

As the second strategy, to identify risk HLAs associated with cardiac phenotypes and explore the mechanisms by which HLAs affect immune responses, we sought to identify patients with CS who had risk HLA-II alleles for cardiac phenotypes, focusing on the interactions between cardiac proteins and HLA-II molecules.

HLA-II molecules are involved in the pathogenesis of autoimmune diseases. Moreover, the binding affinity of HLA-II molecules to antigenic peptides determine their immunogenicity^18^. Using a bioinformatics tool, we comprehensively calculated the molecular affinity between HLA-II molecules encoded by heterodimer-haplotypes and cardiac (possibly autoantigenic) proteins in patients with CS. We listed a total of 172 proteins of interest, considering their functional relationship with cardiac phenotypes (left ventricular dysfunction, atrioventricular block, and ventricular tachycardia) or organ-specific expression in the human heart or other organs (Fig. 1b) (Supplemental Table S12).

**Figure 2:**
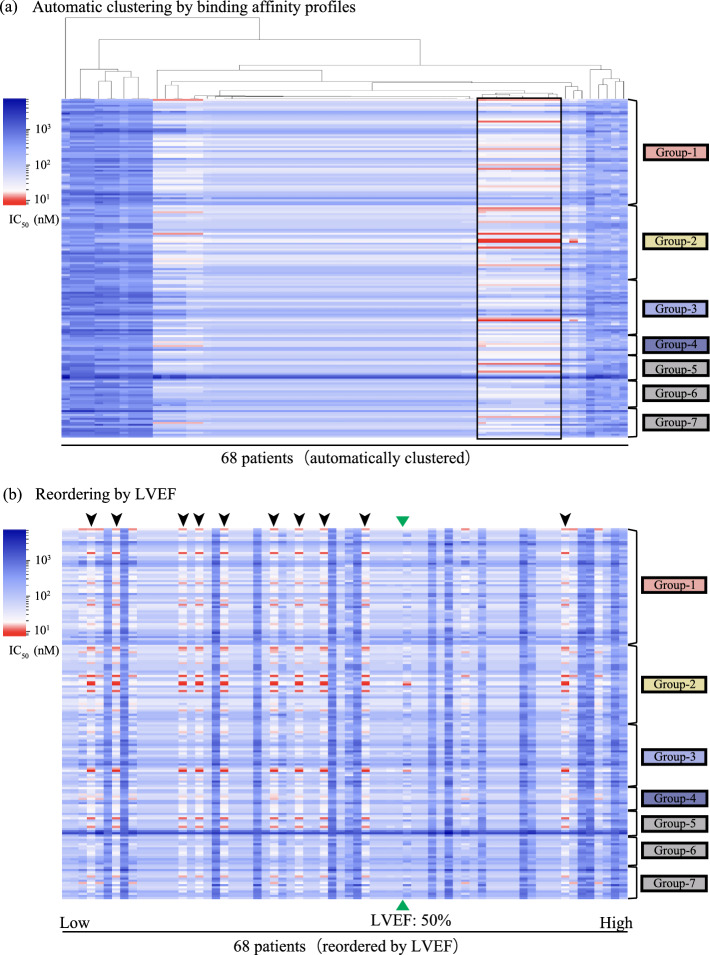
Heatmaps by the minimum IC_50_ profile among 68 patients with CS. The minimum IC_50_ profiles for the HLA-DQ locus among 68 patients with CS were expressed as a heatmap (IC_50_ value above 50 nM were displayed in blue, and IC_50_ values below 50 nM were displayed in two levels of threshold: dark red for IC_50_ value below 12.5 nM, and gradational change from red to white for IC_50_ value above 12.5 nM up to 50 nM) and rearranged in the following manner- (**a**) The profiles were rearranged by hierarchical clustering of affinity profile. The vertical axis is the 172 target proteins to be analysed, grouped by each category. Group-1 to -7 indicate the categories of proteins in Fig. 4. The part surrounded by a square (10 cases) indicates the group with a particularly high BA compared to others. (**b**) The profiles were rearranged in the order of LVEF. The vertical axis is same as Fig. 3a. Cases highlighted by an arrowhead indicate individuals of the group with a particularly high BA in Fig. 3a. The green triangles indicate the borderline of 50% in LVEF.

netMHCIIpan 4.0, a binding affinity calculation algorithm (14) was used to comprehensively calculate binding affinity of every HLA-II molecule expressed in patients with CS (Fig. 1b). We created 15-mer peptide sequences (epitopes) fragmented from the whole amino acid sequences of the proteins by individually shifting the amino acids (Fig. 2a). Then, the binding affinity (IC_50_) of each epitope was calculated individually for all HLA-II heterodimer-haplotypes expressed in patients with CS. Considering the genotype of individuals, each patient has up to two variants of alleles inherited from their father or mother. Each case could theoretically be assumed to have four different haplotypes of HLA-II genes (Supplemental Fig. S1b). To focus on the combinations with the strongest binding affinities, we extracted the combinations with the minimum IC_50_ value among the epitopes from individual proteins. We integrated them as binding affinity profiles for individual cases (Supplemental Fig. S1c). The combinations of HLA-II heterodimer-haplotypes and epitopes with IC_50_ values less than 50 nM were considered strong binding combinations, as reported in previous studies^11^.

### Searching for risk HLA-II heterodimer-haplotypes associated with the low LVEF phenotype using netMHCIIpan

We conducted a de novo study using netMHCIIpan to comprehensively assess the binding affinity between HLA-II and the epitopes from proteins of interest and draw associations between HLA-II and CS phenotypes (Fig. 1b). To identify the epitopes having high affinity towards individual HLA-II heterodimer-haplotypes, we created heatmaps of the binding affinity profiles of epitopes from the 68 patients with CS (Fig. 2). We found numerous patients with similar binding affinity profiles to multiple gene-derived epitopes by using hierarchical clustering (Fig. 2a). In particular, ten cases (the group of cases enclosed in a square in Fig. 2a) exhibited higher affinity for multiple gene-derived epitopes than other patients. These ten cases shared analogous HLA-DQ heterodimer-haplotypes of DQA1*05:0X_DQB1*03:01 (*i.e.*, DQA1*05:03, DQA1*05:05, DQA1*05:06, or DQA1*05:08_DQB1*03:01). These heterodimer-haplotypes were not detected as risk factors in the case–control analysis. To elucidate the common characteristics that these 10 CS cases shared, we explored their cardiac phenotypes. Focusing on the left ventricular systolic dysfunction, we sorted the 68 patients with CS in order of LVEF at enrolment (Fig. 2b). We reported that nine of the ten patients (highlighted by arrowheads in Fig. 2b) exhibited below 50% LVEF at enrolment. Furthermore, no pathogenic variants of the 55 cardiomyopathy-related genes (Supplemental Table S12) were detected in the whole exome sequence data of all nine cases with low LVEF (LVEF < 50%) and DQA1*05:0X_DQB1*03:01 (Table 6). Thus, the low LVEF phenotype in these nine patients with CS was not caused by cardiomyopathy-related gene variants. Through this strategy, we could analyse the possibility of HLA-II involvement of an autoimmune nature in CS pathogenesis.

**Table 6:** Comparison of prevalence of patients with pathogenic variants of cardiomyopathy-related genes among four sub-groups considering left ventricular function and presence of HLA-DQA1*05:0X/DQB1*03:01.

Phenotype	Risk haplotype of DQA1*05:0X /DQB1*03:01	With variants on the CM-related genes (number of patients)	Without variants on the CM-related genes (number of patients)
Low LVEF (LVEF < 50%)	(+)	0	9
	(−)	6	26
Preserved LVEF (LVEF < 51%)	(+)	0	1
	(−)	9	17

### Validity of considering the heterodimer-haplotypes of HLA-DQA1*05:0X_DQB1*03:01 as an group of HLA-DQ haplotypes

**Figure 3:**
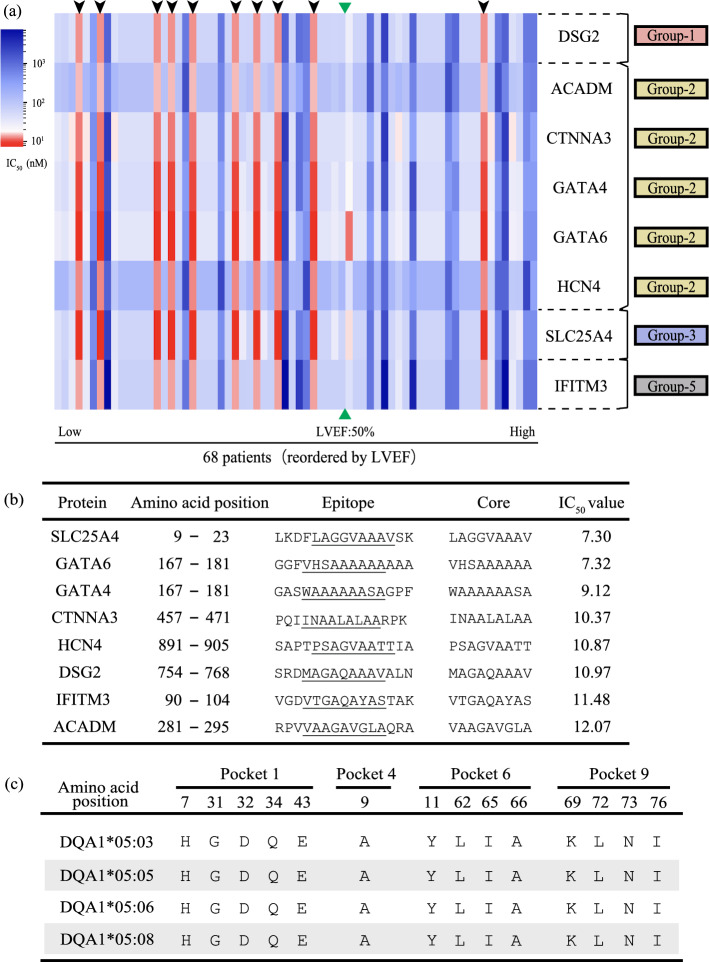
Characteristics of HLA-II heterodimeric haplotypes and proteins with strong binding affinity. (**a**) Heatmap consisting of proteins with strong (IC_50_ < 12.5 nM) BA only at HLA-DQ locus (for 68 patients with CS). The horizontal axis shows the patients with CS arranged in the order of LVEF. The cases highlighted by arrowheads indicate individuals of the group with a particularly high BA in Fig. 2a. These cases harboured the haplotypes DQA1*05:0X/DQB1*03:01. Group-1 to -4 indicate the categories of proteins from Fig. 4. The green triangles indicate the borderline of 50% in LVEF. (**b**) Epitopes with especially strong binding affinities for HLA-DQA1*05:0X/DQB1*03:01 in each protein. The column labelled “Core” indicates the core binding region in each epitope, which is the nine amino acid sequence corresponding to pocket-1 to -9 of the epitope-binding domain. (**c**) The homology of amino acid sequences in anchor pockets of HLA-DQA1*05:0X.

**Figure 4:**
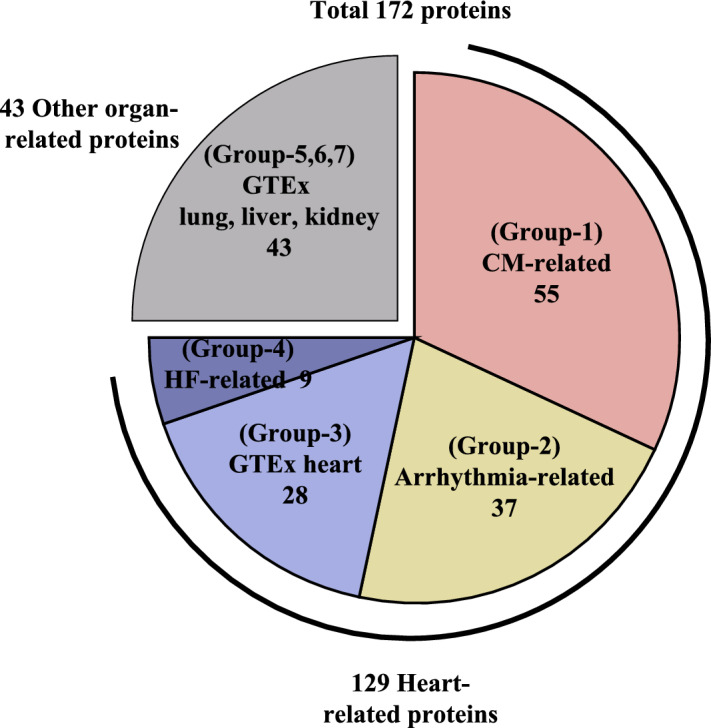
Proteins listed for binding affinity calculation. A total of 172 proteins, comprising 129 heart-related proteins and 43 other (lung, liver, and kidney) organ-related proteins, were listed for binding affinity calculation. Among the heart-related proteins, there were 55 cardiomyopathy-related proteins (CM-related; Group-1), 37 atrioventricular block or ventricular tachycardia-related proteins (Arrhythmia-related; Group-2), 28 proteins derived from genes with high mRNA expression in human heart by GTEx (GTEx heart; Group-3), and nine heart failure-related proteins that the authors considered important (HF-related; Group-4). Among the 43 other organ-related proteins, we identified proteins derived from genes with high mRNA expression in human lung, liver, and kidney using GTEx (GTEx lung, liver, or kidney; Group-5, -6, or -7, respectively).

Multiple HLA molecules have been grouped into HLA supertype^19,20^) based on their binding affinity to antigenic peptides. There have also been attempts of in silico-based HLA supertype classification based on the binding affinity characteristics or amino acid sequences of HLA-II proteins, especially in the epitope-binding domains^18–22^. Supertype classification is an approach for reducing the complexity of HLA classification by reassorting multiple HLA haplotypes into a relatively large group. Thus, we tested whether it was possible to classify the four heterodimer-haplotypes of DQA1*05:0X/DQB1*03:01 into one group of HLA-DQ heterodimeric haplotypes. The HLA-DQ molecules expressed from DQA1*05:0X/DQB1*03:01 heterodimer-haplotypes in the ten patients (highlighted by arrowheads in Figs. 2b, 3a) were found to have the highest affinity for the eight epitopes (Figs. 3a,b). The epitope with high affinity (IC_50_ < 12.5 nM) in each of the eight proteins was identical among the ten cases, regardless of which of the four haplotypes (*i.e*., DQA1*05:03, DQA1*05:05, DQA1*05:06, or DQA1*05:08/DQB1*03:01) they harboured. That is, the HLA molecules expressed from these four heterodimer-haplotypes were predicted to bind strongly to the common epitopes from the eight proteins. The composition of the eight proteins indicated that majority of the proteins were related to cardiac phenotypes (Group-1 or -2 in Fig. 4) or highly expressed in the human heart (Group-3 in Fig. 4). This result suggested that HLA-DQA1*05:0X/DQB1*03:01 molecules might recognise these cardiac proteins as autoantigens and trigger an autoimmune reaction.

We next examined similarity in binding affinity characteristics among the four HLA heterodimer-haplotypes. A previous X-ray crystallography study reported that five important pockets (*i.e*., anchor pockets) in epitope-binding domains of HLA-II molecules (*i.e*., pocket-1, -4, -6, -7, and -9) determined their binding affinity to various epitopes. Moreover, the positions of amino acids forming these pockets in α- and β-chains were also identified (Fig. 1c)^23^. Thus, we checked amino acid residues in the anchor pockets of the four HLA-DQ molecules. We found that they were completely identical among the α-chains expressed from the four heterodimer-haplotypes (DQA1*05:03, DQA1*05:05, DQA1*05:06, or DQA1*05:08/DQB1*03:01) (Fig. 3c). Although pocket 7 is also involved in determining binding affinity, this pocket is formed only by the amino acid residues of the β-chain^24^. Based on these findings, we felt that the four HLA-DQ heterodimer-haplotypes could be grouped into an group of HLA-DQ heterodimeric haplotypes.

Notably, unlike the HLA single alleles and HLA heterodimer-haplotypes detected previously, the correlation of this HLA-DQ heterodimeric haplotypes to cardiac phenotype was unique. We investigated the association between this HLA-DQ heterodimeric haplotypes (DQA1*05:0X/DQB1*03:01) and the cardiac phenotype of low LVEF. The ratio of patients with low LVEF was significantly higher in patients with this HLA-DQ heterodimeric haplotypes than in those without this heterodimeric haplotypes (Table 7). In other words, the presence of this HLA-DQ heterodimeric haplotypes could be associated with low LVEF in patients with CS. HLA-DP or -DR haplotypes were examined similarly; nonetheless, no significant haplotypes were identified. In summary, by clustering multiple HLA-DQ heterodimer-haplotypes based on their binding affinity and pocket structures of epitope-binding domains, we could identify an HLA-DQ heterodimeric haplotypes that was significantly associated with low LVEF in patients with CS.

**Table 7:** Association analysis between the HLA-DQ haplotype, DQA1*05:0X/DQB1*03:01, and clinical phenotypes.

	With DQA1*05:0X/DQB1*03:01 (n = 10)	Without DQA1*05:0X/DQB1*03:01 (n = 58)	P
Clinical characteristics			
Age of onset (years)	61 [56–66]	62 [53–70]	0.75
Gender (female)	6 (60%)	37 (64%)	1.00
Clinical phenotypes			
CAVB	4 (40%)	26 (45%)	1.00
VT or VF	4 (40%)	21 (36%)	1.00
Sustained VT or VF	2 (20%)	15 (26%)	1.00
LVEF (%)	40 [29–46]	47 [36–60]	0.099
Low LVEF	9 (90%)	32 (55%)	0.043 *
Cardiac device implantation	9 (90%)	40 (69%)	0.26
Successful corticosteroid therapy	1 (11%)	20 (43%)	0.13

*CAVB* complete atrioventricular block, *VT* ventricular tachycardia, *VF* ventricular fibrillation, *LVEF* left ventricular ejection fraction, *Low LVEF* the patients with LVEF < 50% in the echocardiography at the enrolment.

*p < 0.05.

### Search for foreign epitopes sharing homology with the autologous epitopes having high binding affinity to the risk HLA-II heterodimer-haplotypes

Thus far, we identified the HLA-DQ heterodimeric haplotypes. However, we thought it necessary to examine the kinds of epitopes recognised by this heterodimeric haplotypes. As CS is an adult-onset disease, we cannot rule out the possibility that some kind of immune activation mechanism from environmental factors was at work in addition to genetic factors. Therefore, we hypothesised that the similarity between foreign- and autologous antigens might activate auto-reactive T-cells in susceptible individuals, and that self-antigen recognition might be acquired through molecular mimicry^11–13^. The mechanism of molecular mimicry requires the presence of foreign antigens with high homology to autologous antigens that have high affinity for the HLA-II heterodimeric haplotypes. Therefore, we used protein BLAST to identify proteins of foreign pathogens (Fig. 1d) that exhibited high homology to the epitopes listed in Fig. 3b.

**Figure 5:**
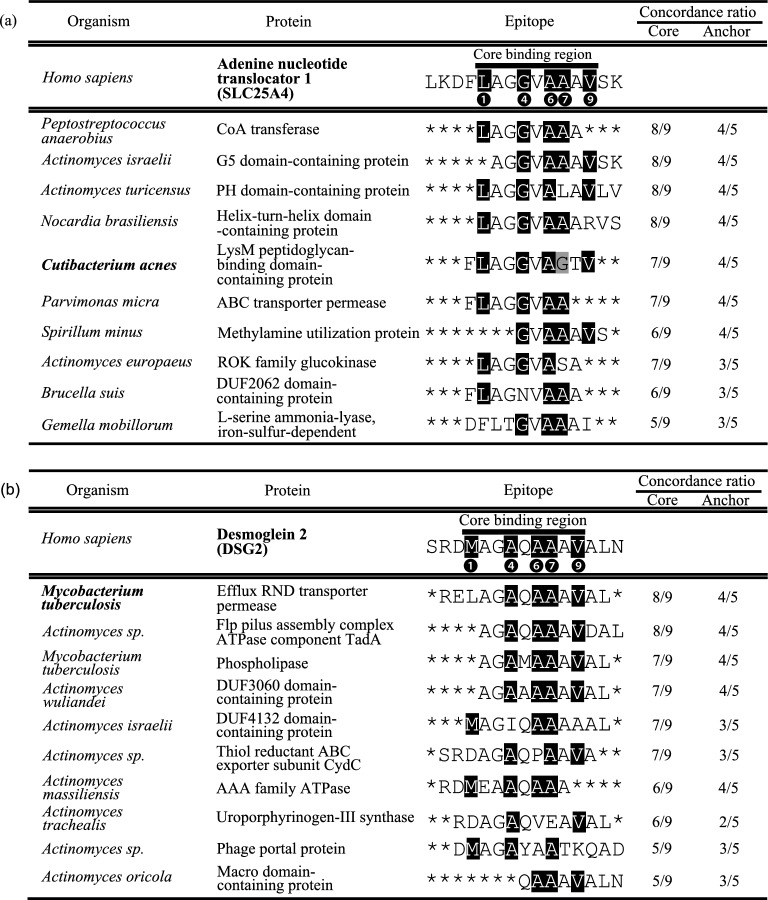
Exogenous pathogen-derived epitopes that are highly homologous to the high affinity epitope of autologous proteins. (**a**) The homology between the epitope “LKDFLAGGVAAAVSK” of SLC25A4, which exhibited the strongest BA for the HLA-DQ heterodimeric haplotypes and proteins from the bacteria which could infect humans were evaluated. The homology was estimated by the concordance ratio at core binding regions predicted by NetMHCIIpan-4.0 or anchor residues (the amino acids of position 1, 4, 6, 7, and 9 in the core binding regions) in each epitope. (**b**) The homology between the epitope “SRDMAGAQAAAVALN” of DSG2, which showed the 6th strongest BA for the HLA-DQ heterodimeric haplotypes and proteins from the bacteria which could infect humans were evaluated. The homology was estimated as in Fig. 5a. The 10 epitopes with the highest concordance ratios are shown.

For each of the eight epitopes (Fig. 3b), we searched for peptides with high peptide homology among the proteins expressed by 160 bacteria that could infect humans. Importantly, five of the nine pockets (pocket-1, -4, -6, -7, and -9) in the epitope-binding domain, corresponding to the anchor residues of epitopes, have key roles in determining the binding affinity (Fig. 1c)^23^. Thus, we evaluated the homology between each of the eight epitopes and the bacterial epitopes by the concordance ratio at core binding regions or anchor residues. We extracted one peptide with the highest homology to the eight epitopes in each bacterium and analysed the concordance ratio (Fig. 5, Supplemental Figs. S2–7).

The epitope “LKDFLAGGVAAAVSK” from human SLC25A4 protein, exhibited the highest binding affinity to HLA-DQA1*05:0X/DQB1*03:01 of the eight epitopes (Fig. 3b). We therefore listed the foreign antigen-derived epitopes in order of homology to the SLC25A4-derived autoantigenic epitope (Fig. 5a). For example, a peptide “FLAGGVAGTV” in Lysin Motif (LysM) peptidoglycan-binding domain-containing protein expressed in *C. acnes* was highly homologous to “LKDFLAGGVAAAVSK” of the human SLC25A4 (concordance ratios for the core binding region and the anchor residues were 7/9 and 4/5, respectively). *C. acnes* has been suggested to be associated with the development of sarcoidosis^5^. Conversely, we also screened peptide sequences homologous to that of “FLAGGVAGTV” in LysM peptidoglycan-binding domain-containing protein, expressed in *C. acnes*, among all proteins expressed in humans. The peptide “FLAGGVAAAV” of the human SLC25A4 was extracted at the top with the same concordance ratio in the core binding region and anchor residues (Supplemental Fig. S8a).

Moreover, the epitope "SRDMAGAQAAAVALN" from human DSG2 exhibited high homology with the epitope “RELAGAQAAAVAL” from Efflux RND transporter permease expressed in *M. tuberculosis*. They exhibited high homology with the core binding region and anchor residues, with concordance ratios of 8/9 and 4/5, respectively (Fig. 5b). Similar to *C. acnes*, *M. tuberculosis* is another bacterium that has been reported to be associated with sarcoidosis incidence^24–27^. Similarly, we screened all proteins expressed in humans for peptide sequences homologous to the "RELAGAQAAAVAL" epitope of *M. tuberculosis* and found that the peptide "RDMAGAQAAAVAL" of human DSG2 may be a highly specific homologous sequence (Supplemental Fig. S8b). In summary, using protein BLAST and accounting for the concordance ratio of core binding regions/anchor residues, we detected foreign epitopes capable of causing an autoimmune reaction via molecular mimicry from bacteria that have been reported to be associated with sarcoidosis incidence.

## Discussion

### Identification of risk HLA-DQ heterodimeric haplotypes for a CS phenotype

Conventionally, associations of HLA alleles with disease incidence have been investigated through single allele-based case–control analyses of autoimmune diseases. However, it is unclear how HLA alleles influence the pathogenesis of autoimmune diseases. Here, we analysed HLA-II haplotypes associated with CS phenotypes, focusing on the heterodimeric structure of HLA-II molecules and their binding to potentially immunogenic epitopes. Of note, comprehensive calculations of binding affinities of HLA-II with candidate autoantigenic epitopes enabled us to detect a group of risk HLAs associated with low LVEF. Moreover, these HLAs could be grouped into an HLA-DQ heterodimeric haplotypes. These results suggest that the heterodimeric haplotypes—HLA-DQA1*05:0X/DQB1*03:01—might increase the risk of left ventricular systolic dysfunction in patients with CS. When grouping multiple heterodimer-haplotypes into a heterodimeric haplotypes, we considered that the anchor pockets in the epitope-binding domain of HLA-II molecules could determine their binding affinity. We proposed that these pockets might play a pivotal role in cross-reactivity of HLA-II molecules with foreign and autologous antigenic epitopes. This could lead to autoimmune-like reactivity via molecular mimicry.

### Risk HLAs affecting disease development and phenotypic expression

In this study, we identified three heterodimer-haplotypes (i.e., DQA1*03:03/DQB1*06:01, DQA1*01:03/DQB1*04:01, and DPA1*02:02/DPB1*09:01) as risk factors associated with CS incidence. However, NetMHCIIpan analysis, which estimates the binding affinity between HLA-II molecules and epitopes of candidate proteins, failed to detect these heterodimer-haplotypes. In contrast, the binding affinity analysis identified four heterodimer-haplotypes, belonging to the HLA-DQ heterodimeric haplotypes (i.e., HLA-DQA1*05:0X/DQB1*03:01), that could not be detected in case–control comparisons. This discrepancy is due to the use of two different analysis methods; The first strategy is for identifying the risk factors of CS susceptibility, while the second strategy is for identifying the exacerbation factors of CS. However, there might be other reasons. The 68 cases in the CS group and 311 cases in the control group used for the case–control comparison did not cover all HLA-II heterodimer-haplotypes, and the number of detected haplotypes were not large enough to test statistically significant differences. Moreover, the 172 candidate proteins used in the binding affinity analysis are not an exhaustive list of all molecules expressed in the heart. Furthermore, we used a very strict cut-off value for the estimated IC_50_ value calculated by NetMHCIIpan (12.5 nM), which may have led us to miss haplotypes due to false negatives. In addition, the present study did not confirm the onset of the disease using standardised criteria, follow-up on trends in echocardiographic indices, nor comprehensively analyse the detection of arrhythmias. These conditions encompass the limitations of this study. Either a validation study or further analysis that account for these limitations may resolve these discrepancies.

### Molecular mimicry induced by antigens derived from foreign pathogens and specificity of cardiac pathology

Since the similarity of pocket structures and statistical analysis revealed that the HLA heterodimeric haplotypes are associated with low LVEF, we investigated how this HLA heterodimeric haplotypes are related to the pathological condition of CS from a biological perspective. We found that HLA-DQA1*05:0X/DQB1*03:01 is analogous with high binding affinity to epitopes from the eight proteins (Fig. 3). Considering the cross-reactivity and immunogenicity of foreign and autologous antigens, epitopes with similar amino acid residues in the anchor pockets of the epitope-binding domain might trigger autoimmune reactions mediated by HLA-II molecules^28,29^. In this study, SLC25A4 and a protein from *C. acnes* share a highly homologous peptide sequence, suggesting that SLC25A4 might trigger autoimmune reactions through molecular mimicry. Furthermore, the peptide sequences of five proteins (i.e., GATA6, GATA4, CTNNA3, DSG2, and IFITM3) were highly homologous to those from *M. tuberculosis* (Fig. 5b, Supplemental Figs. S2-S4, S6).

The SLC25A4 epitope is highly homologous to the "FLAGGVAGTV" sequence from the core binding region and anchor residue of the LysM peptidoglycan-binding domain-containing protein expressed in *C. acnes* (Fig. 5a, Supplemental Fig. S8a), which has been implicated in sarcoidosis incidence. Previous studies showed a B-cell-specific immune response to *C. acnes*^30^, or an increased Th1 immune response and increased IgG and IgA titers in peripheral blood mononuclear cells from sarcoidosis patients with presence of *C. acnes*^31,32^. Bacteria develop a protective response through cell surface immune sensors (pattern recognition receptors, PRRs). The LysM is a PRR and is involved in self- and non-self-recognition. It is known that the amino acid sequences in LysM varies depending on the bacterial species^29,30^. These results might provide clues to understand how exogenous bacteria are involved in the pathogenesis and severity of CS. In other words, in patients with CS with the HLA-DQ heterodimeric haplotypes, *C. acnes* antigens might induce a phenotype of LV systolic dysfunction through molecular mimicry.

In contrast, for proteins from *M. tuberculosis*, epitopes with high homology, especially to the peptide sequence of DSG2, were identified (Fig. 5b, Supplemental Fig. S8b). *M. tuberculosis* has also been reported to be associated with sarcoidosis incidence^24–27^. Some reports have stated that bacteria that infect the hilar lymph nodes via the respiratory tract can spread lymphatically or haematogenously to systemic organs if not eradicated in the lymph nodes, causing infection of various systemic organs and triggering the development of systemic sarcoidosis^32^.

NetMHCIIpan-4.0 we utilized in this study is a bioinformatics tool trained through deep learning with experimental data of binding affinity or ligand eluting assays in IEDB. By utilizing NetMHCIIpan-4.0, we could predict proteins that have not yet been assayed as candidate antigens for pathogenesis. Put another way, epitopes predicted by NetMHCIIpan-4.0 are not always registered in IEDB or underpinned by experimental data. Moreover, we have proposed that we could predict foreign pathogens which could cause auto-immunological reactions in hosts through the peptide homology with the informatics methods described in this study.

Another intriguing thing to note is that proteins from several bacteria belonging to *Actinomyces* spp., a well-known oral commensal, exhibited high homology to the peptide sequences of all eight proteins (Fig. 5, Supplemental Figs. S1-S6). *Actinomyces* spp. are known to cause pulmonary actinomycosis, the primary pathogenesis of which is chronic inflammatory granulomatous lung disease^32,33^. Even exposure levels that do not cause *Actinomyces* infections might elicit inflammatory responses in patients with risk HLA haplotypes that can cause cross-reactivity.

The frequency of HLA-DQA1*05:0X/DQB1*03:01 in the Japanese population is estimated to be 8.1%^34,35^. Haplotype frequency did not differ significantly between the patient group and the control group (14.7% (10/68) and 16.4% (51/311) (p = 0.86) respectively). Thus, these results suggest that genetic factors alone do not cause CS, but that differences in independent environmental factors (e.g., degree of exposure to certain foreign antigens, such as *C. acnes*, *M. tuberculosis*, *Actinomyces* spp., etc.) might define whether individuals with risk HLAs develop CS.

Thus, clinical trials should be conducted to test whether individuals who carry the heterodimeric haplotypes and are exposed to exogenous antigens, such as *C. acnes,* exhibit increased risk of developing CS.

In conclusion, by focusing on the molecular structure of HLA-II and comprehensively analysing the binding affinity between HLA-II molecules and autologous peptides, we were able to identify a group of HLA-II heterodimeric haplotypes that may be associated with CS. We also investigated exogenous antigens with amino acid sequences homologous to the autologous peptides and with high affinity for the at-risk HLA-DQ heterodimeric haplotypes. The results of this study may facilitate the stratification of patients with severe prognosis.

### Limitation of the study

This study is not based on in vitro or in vivo analyses but entirely on the results of the algorithms and bioinformatics analyses. Therefore, the epitopes we detected are hypothetical or theoretical ones and it has not been confirmed whether these epitopes are really generated in the disease. In this study, the binding affinity of epitopes, generated from the amino acid sequences of 172 candidate proteins, to the HLAs of the patients with cardiac sarcoidosis was evaluated using bioinformatics methods. Moreover, the homologies between heart and microbial proteins are also bioinformatically determined and limited to small number of human proteins. To confirm that the epitope we detected elicit T cell responses through molecular mimicry, we need additional studies, in vitro or in vivo, to demonstrate that the epitopes really stimulate T cell response through the mechanism of molecular mimicry and cause disease. Such studies might include experiments as T cell recognition of their peptides and generation of antibody or a T cell receptor that could cross-react to both of foreign and autologous epitopes or purifying the peptides from the MHC in cardiac sarcoidosis using biological samples of the patients with CS, such as bronchoalveolar lavage (BAL) cells or tissue from the heart in left ventricular assist device (LVAD) or heart transplantation procedures to determine the presence of the epitopes on MHC.

We considered it was more promising to focus on *C. acnes* and *M. tuberculosis* epitopes to prove our hypothesis of cross-reactive HLA-II mediated auto-immunogenicity because they have already been implicated in the development of sarcoidosis. Broadening the types of foreign pathogen epitopes included in the study might yield more epitopes that show high homology to the autologous epitopes. Furthermore, this study is a retrospective study in Japanese patients with CS. Prospective validations of the risk HLA haplotypes identified in other Japanese cohorts are necessary. In addition, validation study in other races and ethnicities are necessary, considering the racial differences in HLA allele distribution.

## Methods

### Patients and controls

This study included 68 patients with CS enrolled at five medical centres in Japan. The medical records of patients were reviewed independently by two cardiologists, and only those patients who met the diagnostic criteria (2016 Japanese Circulation Society expert consensus criteria for diagnosis of CS) were enrolled. We obtained genotype information, including both HLA allele typing and whole exome sequencing data. We obtained phenotype and clinical information from the medical records at each institution.

This study was conducted in accordance with the Declaration of Helsinki, and was approved by the Institutional Review Board of Osaka University (accession number: 680) prior to participant enrolment. We also recruited 311 healthy individuals (aged 40–92 years old and without any previous medical history) from Ishikawa prefecture, Japan as controls. They were provided with a self-administered questionnaire and requested to undergo a comprehensive health examination. It was confirmed that they were not suffering from any cardiovascular diseases. Written informed consent was obtained from all the participants.

### Genotyping

#### NGS HLA typing

Eleven classical HLA genes (HLA-A, -B, -C, -DRB1, -DRB3, -DRB4, -DRB5, -DPA1, -DPB1, -DQA1, and -DQB1) were analysed for HLA typing according to AllType NGS 11-Loci Amplification Kit (One Lambda, Los Angeles, CA, USA). The prepared genome libraries were sequenced as 150-bp paired end runs on MiSeq systems (Illumina, San Diego, CA, USA). The typing of six-digit HLA alleles was conducted in TypeStream Visual NGS Analysis Software (Thermo Fisher Scientific, Waltham, MA, USA) with IPD-IGMT/HLA Database release 3.21.0.

#### Pathogenic variant detection in whole exome sequencing data

Variants that met the following criteria were extracted from the variant list of 68 cases with CS obtained by whole exome sequencing and considered pathogenic variants—(i) exonic and non-synonymous variants in RefGene and known gene databases, (ii) variants with minor allele frequency below 0.005 in HGVD and ToMMo3.5k databases, and (iii) variants classified as disease-causing mutations in the Human Gene Mutation Database (HGMD) database or “pathogenic/likely pathogenic” in the standards and guidelines for the interpretation of sequence variants from the American College of Medical Genetics and Genomics and the Association for Molecular Pathology^36^. For more information on whole exome sequencing, see supporting information.

### HLA association analysis

#### Genotyping

With the results of HLA typing, HLA alleles identified in patients in the CS or control groups were listed, and the allele frequency of each allele was calculated in each group. The HLA alleles identified in the CS group were examined using Fisher’s exact test to compare the allele frequency. The number of alleles detected on each locus was used to perform multiple test correction using the Bonferroni method. For the HLA-DP or DQ loci, we compared not only allele frequency, but also heterodimer-haplotype prevalence. As the HLA-DRA1 gene is not polymorphic, we did not subject it to this analysis.

#### Phenotyping

Focusing on left ventricular systolic function at the time of enrolment, we defined “low LVEF” as patients with below 50% echocardiographic LVEF (Teichholz method). Patients with over 50% LVEF were defined as “preserved LVEF”. All patients with CS were divided into the two groups, and the allele frequency and haplotype prevalence in each subgroup were compared using Fisher’s exact test and the Bonferroni method. Patients who developed CAVB at any time after diagnosis during the follow-up period were defined as CAVB positive, while the remaining patients were defined as CAVB negative. We also divided patients with CS into the following two groups: those with and without sustained ventricular tachycardia (sus-VT; sustained premature ventricular contractions that last longer than 30 s or require emergency treatment because of serious symptoms within 30 s) or ventricular fibrillation (VF). The subgroup analyses for CAVB or VT/VF were conducted in the same manner as those for LVEF.

### Bioinformatic calculation of immunodominant peptides

The computation tool, the netMHCIIpan-4.0 server, was used to calculate the binding affinity of 15-mer peptide sequences generated from whole amino acid sequences of targeted proteins to each HLA-II haplotype (molecule) expressed in each CS patient. A total of 172 proteins, translated from cardiomyopathy-related genes (55 genes; Group-1 in Fig. 4), arrhythmia (AVB or VT)-related genes (37 genes; Group-2 in Fig. 4), genes with high mRNA-level expression in human left ventricle (28 genes; Group-3 in Fig. 4), heart failure-related genes (nine genes; Group-4 in Fig. 4) and genes with high mRNA-level expression in human lung, liver, or kidney (total: 43 genes; Group-5, -6, or -7, respectively) were listed as targeted proteins. We adopted the genes listed in the scientific statement from the American Heart Association for genetic testing of heritable cardiovascular diseases^20^ and the guidelines for diagnosis and treatment of cardiomyopathies from the Japanese Circulation Society^37^ as cardiomyopathy-related genes. We searched for genes in the HGMD or Human Phenotype Ontology with the keywords “atrioventricular block” or “ventricular tachycardia” and constructed a set of arrhythmia-related genes. We then excluded genes which had not been reported to be associated with AVB or VT (searched for in PubMed). We included nine genes as heart failure-related genes. The top 50 genes expressed in each organ were searched for in the GTEx portal (https://gtexportal.org/home/) to produce a list of genes with high mRNA expression in the left ventricle, lung, liver, and kidney. The lists of proteins in each group were described in Supplemental Table S11. We adopted the IC_50_ value as an indicator of binding affinity, and defined combinations (HLA molecules and epitopes) with IC_50_ values less than 50 nM as strong binding combinations.

### Clustering multiple HLA haplotypes into a group of HLA heterodimeric haplotypes

We made a heatmap using the calculated binding affinity data (IC_50_ values) of each epitope from each protein to HLA-II molecules expressed in each patient with CS to visualise the affinity profiles between each HLA-II and each protein. We compared the amino acid residues of anchor pockets (pocket-1, -4, -6, -7, and -9) in the epitope-binding domain of HLA-II with similar affinity profiles. HLA-II molecules with the same amino acid residues in all five pockets were classified into a group of HLA heterodimeric haplotypes.

### Analyses of homology between autoantigenic epitopes and foreign peptide sequences

For each epitope that was calculated to have high affinity for HLA-II, we searched for peptides of high homology among the proteins expressed in 160 bacteria that could infect humans, extracted with reference to the Sanford Guide^38^. For each bacterium, a representative peptide that was most homologous to the original (autologous) epitope was extracted using protein BLAST (https://blast.ncbi.nlm.nih.gov/Blast.cgi?PAGE=Proteins). We then selected only the peptides with E-values < 1.0 and compared the identity of their core binding regions and anchor residues with those of the original epitopes.

### Supplementary Information

Supplementary Information.

## Data Availability

Nucleotide sequence data of 36 patients with cardiac sarcoidosis analysed in this study are available in the DNA Data Bank of Japan (DDBJ) Sequenced Read Archive under the accession numbers JGAS000568 and JGAD000694 (https://ddbj.nig.ac.jp/resource/jga-study/JGAS000568). The sequence data of the remaining 32 patients are going to be released after the consent for registration in public databases is obtained. After approval of a proposal, our data are going to be shared only for non-profit research purposes (https://www.ddbj.nig.ac.jp/index-e.html).
