# Supplementary material for: Human leukocyte antigen-DQ risk heterodimeric haplotypes of left ventricular dysfunction in cardiac sarcoidosis: an autoimmune view of its role
Source: Sci Rep. 2023 Nov 13;13:19767. doi: 10.1038/s41598-023-46915-1 (PMC10643531; doi:10.1038/s41598-023-46915-1)
Supplement: Supplementary file 1 — Supplementary Information. [file 41598_2023_46915_MOESM1_ESM.pdf]

Supplemental figure S1

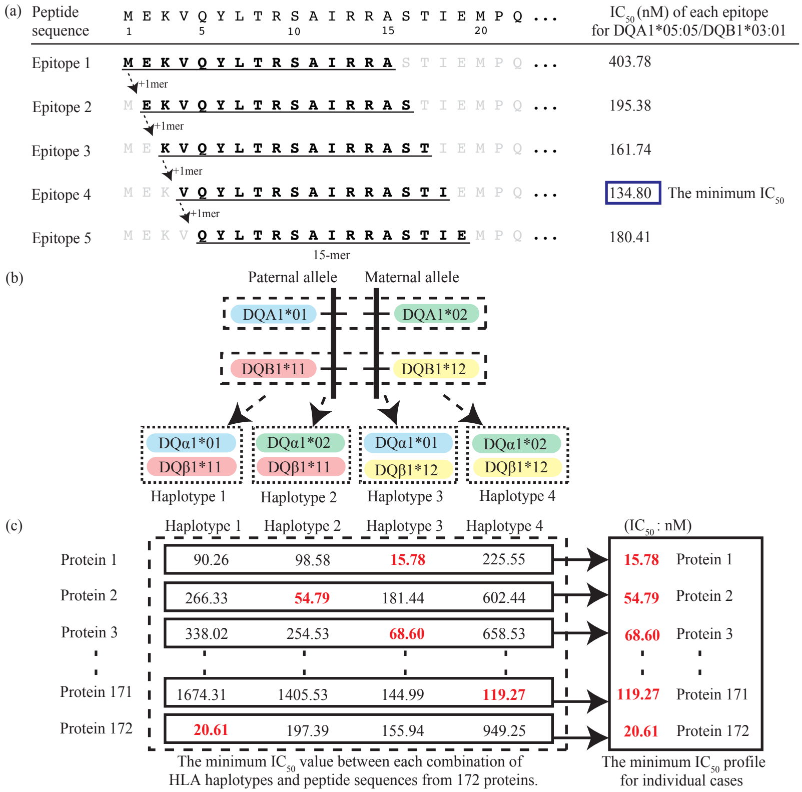

Supplemental figure S1. Algorithm of the binding affinity prediction between HLA class II haplotypes and fragmented peptide sequences (a) Evaluation model of the binding affinity (IC50) using phospholamban protein and HLA-DQA1*05:05/DQB1*03:01 haplotype as an example. Binding affinity between HLA-II haplotypes and each protein-derived epitope was calculated in the following manner—binding affinity of 15-mer amino acid sequences selected from each protein and each HLA-II haplotype was calculated. Binding affinity was comprehensively evaluated by one-by-one shifting of the 15-mer amino acid sequence frame. Then, among all the combinations, the 15-mer with the highest binding affinity was chosen as "the minimum IC$_{50}$ value" for its HLA haplotype and protein; (b) Schematic illustration of the binding affinity profile in each individual. An individual could express up to four combinations of HLA haplotypes in each class II locus considering the heterogenicity of genotypes; (c) Comprehensive evaluation and listing of the minimum IC50 value among four types of haplotypes and 172 targeted proteins in each class II locus. Subsequently, the minimum IC50 profile for each individual was created by extracting the minimum values of IC50 for each protein.

Supplemental figure S2

Organism	Protein	Epitope	Concordance ratio	
			Core	Anchor
Homo sapiens	**GATA binding protein 6 (GATA6)**	Core binding region<br>GGFVHSAAAAAAAAA<br>❶ ❹ ❻❼ ❾		
Anaplasma phagocytopilium	DUF4401 domain-containing protein	****HSTAAAAAAAA	8/9	3/5
Burkholderia cepacia	N-acetylglucosamine-specific PTS transporter	GGFVAPAAAAAAAA*	7/9	5/5
Mycobacterium tuberculosis	NAD(P)H-hydrate dehydratase	**FVHARAAAAAAAA	7/9	4/5
Haemophilus ducreyi	50S ribosomal protein L7/L12	*****SAAAAVAAAA	7/9	4/5
Burkholderia multivorans	Sugar transporter	****HAAAAAAAAAA	7/9	4/5
Haemophilus influenzae	Porin	*****SAAAAAAAAA	7/9	4/5
Actinomyces israelii	S8 family serine peptidase	GGFDTSAAAATFAAA	7/9	3/5
Rickettsia	DUF4401 domain-containing protein	****HSTAAAAAAAA	7/9	3/5
Brucella canis	P-type conjugative transfer protein VirB9	******AAAAAAAAA	6/9	4/5
Brucella suis	C-type cytochrome	***QNAAAAAAAAA*	6/9	4/5
Chlamydia pneumoniae	Molecular chaperone DnaK	****QSASAAAASAA	6/9	4/5
Helicobacter cinaedi	Nitrate reductase catalytic subunit	**FIKSAAAASAAA*	6/9	4/5
Actinomyces meyeri	Serine/threonine protein kinase	****HAAAAPAASAA	6/9	3/5
Nocardia asteroides	DUF1906 domain-containing protein	AAFVGAAAAA*****	5/9	4/5

Supplemental figure S2. Exogenous pathogen-derived epitopes that are highly homologous to the high affinity epitope of GATA6 protein

The homology between the epitope "GGFVHSAAAAAAAAA" of GATA6 protein, which exhibited the second strongest binding affinity for the HLA-DQ supertype (DQA1*05:0X/DQB1*03:01) and proteins from the bacteria which could infect humans were evaluated. The homology was estimated based on concordance ratio at core binding regions (9-mer peptide sequences predicted by netMHCIIpan-4.0) or anchor residues (the amino acids of position 1, 4, 6, 7, and 9 in the core binding regions) in each epitope.

Supplemental figure S3

Organism	Protein	Epitope	Concordance ratio	
			Core	Anchor
Homo sapiens	**GATA binding protein 4 (GATA4)**	Core binding region<br>GASWAAAAAASAGPF<br>❶ ❹ ❻❼ ❾		
Actinomyces sp.	Stk1 family PASTA domain-containing Ser/Thr kinase	**SWAAAGAASA***	8/9	5/5
Actinomyces sp.	TetR family transcriptional regulator	****AAAAAASAAP*	8/9	4/5
Actinomyces glycerinitolerans	LamG domain-containing protein	**SWAAQTAASAAP*	7/9	4/5
Actinomyces sp.	Preprotein translocase subunit SecA	**GAAAAAAAAAGP*	7/9	4/5
Actinomyces sp.	AMP-binding protein	****AVAAAASAGP*	7/9	4/5
Actinomyces dentalis	Glycosyltransferase	GGTWAAAAAGSTG**	7/9	3/5
Actinomyces israelii	Fructosamine kinase family protein	**SWAAAASDSASAG	7/9	3/5
Mycobacterium tuberculosis	PPE family protein	GASWAGPAAAS****	6/9	3/5
Mycobacterium tuberculosis	PE family protein	GAGWYAAAEAASA**	5/9	3/5
Actinomyces sp.	DUF1156 domain-containing protein	GGSWPEIAAAARTAA	4/9	3/5

Supplemental figure S3. Exogenous pathogen-derived epitopes that are highly homologous to the high affinity epitope of GATA4 protein

The homology between the epitope "GASWAAAAAASAGPF" of GATA4 protein, which exhibited the third strongest binding affinity for the HLA-DQ supertype (DQA1*05:0X/DQB1*03:01) and proteins from the bacteria which could infect humans were evaluated. The homology was estimated based on concordance ratio at core binding regions (9-mer peptide sequences predicted by netMHCIIpan-4.0) or anchor residues (the amino acids of position 1, 4, 6, 7, and 9 in the core binding regions) in each epitope.

Supplemental figure S4

Organism	Protein	Epitope	Concordance ratio	
			Core	Anchor
Homo sapiens	**Catenin alpha 3 (CTNNA3)**	Core binding region: PQIINAALALAARPK (❶ ❹ ❻❼ ❾)		
Actinomyces slackii	Na^+/H^+ antiporter NhaA	PEAINAALALEA***	8/9	5/5
Mycobacterium tuberculosis	Phosphate ABC transporter permease subunit PstC	LIQIEAALALAA***	8/9	5/5
Actinomyces polynesiensis	DeoR/GlpR family DNA-binding transcription regulator	***INAALARAAR**	7/9	4/5
Mycobacterium tuberculosis	Pyridoxal-phosphate dependent enzyme	*****AALALAAR**	7/9	4/5
Actinomyces sp.	NAD/FAD-dependent oxidoreductase	*****AALALAARP*	7/9	4/5
Actinomyces sp.	ATP-binding cassette domain-containing protein	******ALALAARP*	6/9	4/5
Mycobacterium tuberculosis	3’ 5’ -biphosphate nucleotidase CysQ	PEITDAAVALPAR**	5/9	4/5
Actinomyces sp.	S8 family serine peptidase	**IINAAAAVTELAA	5/9	3/5
Actinomyces dentalis	MarR family transcriptional regulator	**IINAELTALAAMP	5/9	2/5
Mycobacterium tuberculosis	Phage tail tape measure protein	*QIIGGSLNGALALA	3/9	1/5

Supplemental figure S4. Exogenous pathogen-derived epitopes that are highly homologous to the high affinity epitope of CTNNA3 protein

The homology between the epitope “PQIINAALALAARPK” of CTNNA3 protein, which exhibited the forth strongest binding affinity for the HLA-DQ supertype (DQA1*05:0X/DQB1*03:01) and proteins from the bacteria which could infect humans were evaluated. The homology was estimated based on concordance ratio at core binding regions (9-mer peptide sequences predicted by netMHCIIpan-4.0) or anchor residues (the amino acids of position 1, 4, 6, 7, and 9 in the core binding regions) in each epitope.

Supplemental figure S5

Organism	Protein	Epitope	Concordance ratio	
			Core	Anchor
Homo sapiens	**Potassium/sodium hyperpolarization-activated cyclic nucleotide-gated channel 4 (HCN4)**	Core binding region<br>SAPTPSAGVAATTIA<br>❶ ❹ ❻❼ ❾		
Actinomyces sp.	Iron-siderophore ABC transporter substrate-binding protein	SASTASAGVAAQTTA	7/9	4/5
Actinomyces massiliensis	LysE/ArgO family amino acid transporter	SAARPAASVAATTLA	7/9	4/5
Actinomyces oris	Byfunctional metallophosphatase/ 5’ nucleotidase	**PTPSTGVATST**	6/9	4/5
Actinomyces naeslundii	Response regulator transcription factor	SPSTPSGGIASTT**	6/9	4/5
Actinomyces sp.	Flippase-like domain-containing protein	*APTPSAGSAST***	6/9	3/5
Actinomyces bowdenii	Amin domain protein	SAPRPSATGSAATT*	5/9	3/5
Actinomyces sp.	DEAD/DEAH box helicase	*SATPSAGAAPRPPA	5/9	3/5
Actinomyces polynesiensis	Translation initiation factor IF-2 N-terminal domain-containing protein	*APTPSAGATA****	5/9	3/5
Actinomyces sp.	Translation initiation factor2	SAPTPSAPVA*****	5/9	2/5
Chlamydia psittaci	Autotransporter domain-containing protein	*APTPAKVAATT***	3/9	2/5

Supplemental figure S5. Exogenous pathogen-derived epitopes that are highly homologous to the high affinity epitope of HCN4 protein

The homology between the epitope “SAPTPSAGVAATTIA” of HCN4 protein, which exhibited the fifth strongest binding affinity for the HLA-DQ supertype (DQA1*05:0X/DQB1*03:01) and proteins from the bacteria which could infect humans were evaluated. The homology was estimated based on concordance ratio at core binding regions (9-mer peptide sequences predicted by netMHCIIpan-4.0) or anchor residues (the amino acids of position 1, 4, 6, 7, and 9 in the core binding regions) in each epitope.

Supplemental figure S6

Organism	Protein	Epitope	Concordance ratio	
			Core	Anchor
Homo sapiens	**Interferon-induced transmembrane protein 3 (IFITM3)**	Core binding region<br>VGDVTGAQAYASTAK<br>❶ ❹ ❻❼ ❾		
Actinomyces culturomici	Alpha/beta hydrolase	***VTGTQAYA****	7/9	3/5
Actinomyces sp.	DUF1156 domain-containing protein	****TGAQAYA****	7/9	3/5
Mycobacterium tuberculosis	DUF3566 domain-containing protein	*GDVPPAEAYAS***	6/9	5/5
Actinomyces polynesiensis	DNA methylase	**VGDVADAYAS***	5/9	4/5
Cutibacterium acnes	Site-specific DNA-methyltransferase	**VGDVADAYAS***	5/9	4/5
Actinomyces sp.	Undecaprenyl/decaprenyl-phosphate alpha-N-acetylglucosaminyl 1-phosphate transferase	*****GAQSYATTA*	5/9	3/5
Actinomyces sp.	Type II secretion system F family protein	******AQAYATTA*	5/9	3/5
Actinomyces sp.	Integral membrane protein MviN	*GDVTGAQ*******	5/9	2/5
Actinomyces graevenitzii	Fumarylacetoacetate hydrolase family protein	*GDVILTGAAAYA**	3/9	3/5
Actinomyces howellii	ABC transporter permease subunit	******QAEYASTAK	2/9	2/5

Supplemental figure S6. Exogenous pathogen-derived epitopes that are highly homologous to the high affinity epitope of IFITM3 protein

The homology between the epitope “VGDVTGAQAYASTAK” of IFITM3 protein, which exhibited the seventh strongest binding affinity for the HLA-DQ supertype (DQA1*05:0X/DQB1*03:01) and proteins from the bacteria which could infect humans were evaluated. The homology was estimated based on concordance ratio at core binding regions (9-mer peptide sequences predicted by netMHCIIpan-4.0) or anchor residues (the amino acids of position 1, 4, 6, 7, and 9 in the core binding regions) in each epitope.

Supplemental figure S7

Organism	Protein	Epitope	Concordance ratio	
			Core	Anchor
Homo sapiens	**Acyl-CoA Dehydrogenase Medium Chain (ACADM)**	Core binding region<br>RPVVAAGAVGLAQRA<br>❶ ❹ ❻❼ ❾		
Actinomyces sp.	Threonine/serine exporter family protein	***VAAGAGGLARRA	8/9	4/5
Actinomyces bowdenii	Proteasome accessory factor PafA2 family protein	PVMVGAGRVGLGQR*	6/9	4/5
Actinomyces sp.	Glycosyltransferase family 2 protein	RPVVAAGAAG*****	6/9	3/5
Actinomyces provencensis	IS1380 family transposase	***VAAGGLPAVMGL	4/9	2/5
Actinomyces sp.	2-oxo acid dehydrogenase subunit E2	PVVAADGTIGVEQR*	3/9	2/5

Supplemental figure S7. Exogenous pathogen-derived epitopes that are highly homologous to the high affinity epitope of ACADM protein

The homology between the epitope "RPVVAAGAVGLAQRA" of ACADM protein, which exhibited the eighth strongest binding affinity for the HLA-DQ supertype (DQA1*05:0X/DQB1*03:01) and proteins from the bacteria which could infect humans were evaluated. The homology was estimated based on concordance ratio at core binding regions (9-mer peptide sequences predicted by netMHCIIpan-4.0) or anchor residues (the amino acids of position 1, 4, 6, 7, and 9 in the core binding regions) in each epitope.

Supplemental figure S8

(a)

Organism	Protein	Epitope	Concordance ratio	
			Core	Anchor
Cutibacterium acnes	LysM peptidoglycan -binding domain -containing protein	Core binding region<br>FLAGGVAGTV<br>❶ ❹ ❻❼ ❾		
Homo sapiens	SLC25A24	*LAGGIAGAV	7/9	5/5
Homo sapiens	**SLC25A4**	FLAGGVAAAV	7/9	4/5
Homo sapiens	AMN	*LAGGVAAAV	7/9	4/5
Homo sapiens	SLC25A31	*LAGGVAAAV	7/9	4/5
Homo sapiens	SLC25A16	FLAGGIAG**	7/9	4/5

(b)

Organism	Protein	Epitope	Concordance ratio	
			Core	Anchor
Mycobacterium tuberculosis	Efflux RND transporter permease	Core binding region<br>RELAGAQAAAVAL<br>❶ ❹ ❻❼ ❾		
Homo sapiens	DHX34	**LAGAQAAQVAL	8/9	5/5
Homo sapiens	**DSG2**	RDMAGAQAAAVAL	8/9	4/5
Homo sapiens	POU6F1	**LAGLQAAAVLN	8/9	4/5
Homo sapiens	SMAD4	***AAAQAAAVA*	7/9	4/5
Homo sapiens	PRX	****GAQAAAV**	7/9	3/5

Supplemental figure S8. Autologous protein-derived epitopes that are highly homologous to the epitope of foreign pathogens.

(a) The homology between the epitope "FLAGGVAGTV" of LysM peptideglycan-binding domain-containing protein derived from ***Cutibacterium acnes*** and proteins expressed in humans. The homology was estimated based on the concordance ratio in the core binding regions (9-mer peptide sequence predicted by NetMHCIIpan-4.0) oranchor residues (the amino acids of position 1, 4, 6, 7, and 9 in the core binding regions) in each epitope. (b) The homology between the epitope "RELAGAQAAAVAL" of efflux RND transporter permease derived from ***Mycobacterium tuberculosis*** and proteins expressed in humans. The homology was estimated based on concordance ratio in the core binding regions or anchor residues in each epitope. The five epitopes with the highest concordance ratios are shown.

Supplemental table S1. Association analysis for the risk HLA-***DRB1*** allele between CS patients and controls

Allele	Cardiac sarcoidosis (n = 68)	Control (n = 311)	P_adj
DRB1*08:03	29 (43%)	51 (16%)	**1.9E-04** *
DRB1*04:05	22 (32%)	58 (19%)	n.s.
DRB1*01:01	1 (1.5%)	28 (9.0%)	n.s.
DRB1*04:03	1 (1.5%)	21 (6.8%)	n.s.
DRB1*12:01	9 (13%)	24 (7.7%)	n.s.
DRB1*04:10	5 (7.4%)	11 (3.5%)	n.s.
DRB1*08:02	10 (15%)	35 (9.6%)	n.s.
DRB1*04:01	3 (4.4%)	8 (2.6%)	n.s.
DRB1*14:54	1 (1.5%)	12 (3.9%)	n.s.
DRB1*09:01	19 (28%)	100 (32%)	n.s.
DRB1*15:01	8 (12%)	47 (15%)	n.s.
DRB1*13:02	5 (7.4%)	31 (10%)	n.s.
DRB1*15:02	14 (21%)	57 (18%)	n.s.
DRB1*13:01	2 (2.9%)	14 (4.5%)	n.s.
DRB1*04:06	2 (2.9%)	12 (3.9%)	n.s.
DRB1*11:01	2 (2.9%)	12 (3.9%)	n.s.
DRB1*12:02	1 (1.5%)	6 (1.9%)	n.s.
DRB1*14:06	1 (1.5%)	9 (2.9%)	n.s.

* Significance after the correction for multiple testing (Bonferroni correction); P_adj. < 0.05

CS : Cardiac sarcoidosis, n.s.: No significant difference

Supplemental table S2. Association analysis for the risk *HLA-**DQA1*** allele between CS patients and controls

Allele	Cardiac sarcoidosis (n = 68)	Control (n = 311)	P_adj
DQA1*01:03	40 (59%)	117 (38%)	**0.022** *
DQA1*03:03	30 (44%)	93 (30%)	n.s.
DQA1*01:01	1 (1.5%)	28 (9.0%)	n.s.
DQA1*01:04	1 (1.5%)	20 (6.4%)	n.s.
DQA1*01:02	12 (18%)	79 (25%)	n.s.
DQA1*05:05	8 (12%)	22 (7.1%)	n.s.
DQA1*05:06	1 (1.5%)	2 (0.6%)	n.s.
DQA1*06:01	1 (1.5%)	10 (3.2%)	n.s.
DQA1*05:03	1 (1.5%)	11 (3.5%)	n.s.
DQA1*05:08	1 (1.5%)	11 (3.5%)	n.s.
DQA1*03:01	10 (15%)	53 (17%)	n.s.
DQA1*04:01	3 (4.4%)	19 (6.1%)	n.s.
DQA1*03:02	21 (31%)	102 (33%)	n.s.

* Significance after the correction for multiple testing (Bonferroni correction); P_adj. < 0.05

CS : Cardiac sarcoidosis, n.s.: No significant difference

Supplemental table S3. Association analysis for the risk *HLA-A* allele between CS patients and controls

Allele	Cardiac sarcoidosis (n = 68)	Control (n = 311)	P_adj
A*11:01	18 (27%)	36 (12%)	**0.047** *
A*24:02	34 (50%)	196 (63%)	n.s.
A*11:02	2 (2.9%)	1 (0.3%)	n.s.
A*26:02	3 (4.4%)	6 (1.9%)	n.s.
A*02:06	14 (21%)	51 (16%)	n.s.
A*31:01	10 (15%)	57 (18%)	n.s.
A*02:07	6 (8.8%)	21 (6.8%)	n.s.
A*24:20	2 (2.9%)	6 (1.9%)	n.s.
A*03:01	2 (2.9%)	7 (2.3%)	n.s.
A*26:03	1 (1.5%)	11 (3.5%)	n.s.
A*01:01	1 (1.5%)	7 (2.3%)	n.s.
A*02:01	15 (22%)	70 (23%)	n.s.
A*26:01	9 (13%)	44 (14%)	n.s.
A*33:03	9 (13%)	44 (14%)	n.s.

* Significance after the correction for multiple testing (Bonferroni correction); P_adj. < 0.05

CS : Cardiac sarcoidosis, n.s.: No significant difference

Supplemental table S4. Association analysis for the risk HLA-***C*** allele between CS patients and controls

Allele	Cardiac sarcoidosis (n = 68)	Control (n = 311)	P_adj
C*03:04	5 (7.4%)	78 (25%)	**0.016** *
C*01:02	29 (43%)	87 (28%)	n.s.
C*04:82	3 (4.4%)	4 (1.3%)	n.s.
C*03:03	12 (18%)	82 (26%)	n.s.
C*14:02	4 (5.9%)	33 (11%)	n.s.
C*01:03	1 (1.5%)	2 (0.6%)	n.s.
C*15:02	8 (12%)	28 (9.0%)	n.s.
C*07:02	17 (25%)	69 (22%)	n.s.
C*14:03	9 (13%)	35 (11%)	n.s.
C*06:02	1 (1.5%)	10 (3.2%)	n.s.
C*12:02	14 (21%)	68 (22%)	n.s.
C*03:02	1 (1.5%)	6 (1.9%)	n.s.
C*04:01	5 (7.4%)	23 (7.4%)	n.s.
C*05:01	2 (2.9%)	9 (2.9%)	n.s.
C*07:04	1 (1.5%)	4 (1.3%)	n.s.
C*08:01	7 (10%)	34 (11%)	n.s.

* Significance after the correction for multiple testing (Bonferroni correction); P_adj. < 0.05

CS : Cardiac sarcoidosis, n.s.: No significant difference

Supplemental table S5. Comparison of allele prevalence between CS and control groups

	CS		Controls		P_adj.		CS		Controls		P_adj.
	cases	%	cases	%			cases	%	cases	%	
B*07:02	2	2.9	30	9.6	n.s.	DPA1*01:03	38	55.9	192	61.7	n.s.
B*15:01	12	17.6	48	15.4	n.s.	DPA1*02:01	21	30.9	93	29.9	n.s.
B*15:11	2	2.9	10	3.2	n.s.	DPA1*02:02	52	76.5	209	67.2	n.s.
B*15:18	1	1.5	10	3.2	n.s.	DPA1*02:07	1	1.5	1	0.3	n.s.
B*15:28	1	1.5	2	0.6	n.s.	DPB1*02:01	28	41.2	131	42.1	n.s.
B*27:04	2	2.9	1	0.3	n.s.	DPB1*02:02	7	10.3	10	3.2	n.s.
B*35:01	7	10.3	43	13.8	n.s.	DPB1*03:01	2	2.9	22	7.1	n.s.
B*37:01	1	1.5	7	2.3	n.s.	DPB1*04:01	5	7.4	22	7.1	n.s.
B*38:02	2	2.9	2	0.6	n.s.	DPB1*04:02	10	14.7	63	20.3	n.s.
B*39:01	8	11.8	19	6.1	n.s.	DPB1*05:01	50	73.5	198	63.7	n.s.
B*39:02	1	1.5	2	0.6	n.s.	DPB1*06:01	2	2.9	1	0.3	n.s.
B*40:01	8	11.8	32	10.3	n.s.	DPB1*09:01	13	19.1	55	17.7	n.s.
B*40:02	6	8.8	54	17.4	n.s.	DPB1*13:01	1	1.5	7	2.3	n.s.
B*40:06	5	7.4	18	5.8	n.s.	DPB1*135:01	2	2.9	6	1.9	n.s.
B*44:02	2	2.9	9	2.9	n.s.	DPB1*14:01	3	4.4	7	2.3	n.s.
B*44:03	9	13.2	35	11.3	n.s.	DPB1*19:01	1	1.5	1	0.3	n.s.
B*46:01	11	16.2	33	10.6	n.s.	DPB1*36:01	1	1.5	0	0.0	n.s.
B*48:01	2	2.9	18	5.8	n.s.	DRB3*01:01	10	14.7	44	14.1	n.s.
B*48:47	1	1.5	0	0.0	n.s.	DRB3*01:12	1	1.5	0	0.0	n.s.
B*51:01	6	8.8	49	15.8	n.s.	DRB3*02:02	4	5.9	47	15.1	n.s.
B*51:02	1	1.5	2	0.6	n.s.	DRB3*03:01	6	8.8	36	11.6	n.s.
B*52:01	15	22.1	67	21.5	n.s.	DRB4*01:02	3	4.4	9	2.9	n.s.
B*54:01	13	19.1	47	15.1	n.s.	DRB4*01:03	45	66.2	195	62.7	n.s.
B*55:02	5	7.4	11	3.5	n.s.	DRB5*01:01	8	11.8	49	15.8	n.s.
B*56:01	1	1.5	5	1.6	n.s.	DRB5*01:02	14	20.6	62	19.9	n.s.
B*56:03	1	1.5	0	0.0	n.s.						
B*58:01	1	1.5	6	1.9	n.s.						
B*59:01	1	1.5	9	2.9	n.s.						

CS : Cardiac sarcoidosis, n.s.: No significant difference

Supplemental table S6. Associations between the risk HLA allele ***DRB1*08:03*** and clinical characteristics

	**With** DRB1*08:03 (n = 29)	**Without** DRB1*08:03 (n = 39)	P
**Clinical characteristics**			
Age of onset (years)	64 [57-75]	62 [52-57]	0.42
Gender (female)	16 (55%)	27 (69%)	0.31
**Clinical phenotypes**			
CAVB	13 (45%)	17 (44%)	1.00
VT or VF	11 (38%)	14 (36%)	1.00
— Sustained VT or VF	6 (21%)	11 (28%)	0.58
LVEF (%)	48 [35-60]	45 [31-57]	0.41
— Low LVEF	15 (52%)	26 (67%)	0.32
Cardiac device implantation	22 (76%)	27 (69%)	0.60
Successful corticosteroid therapy	12 (48%)	9 (29%)	0.17

CAVB : complete atrioventricular block, VT : ventricular tachycardia, VF : ventricular fibrillation,
Low LVEF: the patients with LVEF < 50% in the echocardiography at their enrollment

* : $p < 0.05$

Supplemental table S7. Associations between the risk HLA allele ***DQA1*01:03*** and clinical characteristics

	**With** *DQA1*01:03* (n = 40)	**Without** *DQA1*01:03* (n = 28)	P
**Clinical characteristics**			
Age of onset (years)	63 [56-71]	59 [51-69]	0.41
Gender (female)	25 (63%)	18 (64%)	1.00
**Clinical phenotypes**			
CAVB	19 (48%)	11 (39%)	0.62
VT or VF	17 (43%)	8 (29%)	0.31
— Sustained VT or VF	11 (28%)	6 (21%)	0.77
LVEF (%)	46 [34-59]	46 [32-62]	0.90
— Low LVEF	3 (60%)	38 (60%)	1.00
Cardiac device implantation	31 (78%)	18 (64%)	0.28
Successful corticosteroid therapy	15 (38%)	6 (21%)	0.39

CAVB : complete atrioventricular block, VT : ventricular tachycardia, VF : ventricular fibrillation,
Low LVEF: the patients with LVEF < 50% in the echocardiography at their enrollment

* : $p < 0.05$

Supplemental table S8. Associations between the risk HLA allele ***HLA-A*11:01*** and clinical characteristics

	**With** *HLA-A*11:01* (n = 18)	**Without** *HLA-A*11:01* (n = 50)	P
**Clinical characteristics**			
Age of onset (years)	66 [55-80]	62 [54-68]	0.34
Gender (female)	12 (67%)	31 (62%)	0.78
**Clinical phenotypes**			
CAVB	8 (44%)	22 (44%)	1.00
VT or VF	10 (56%)	15 (30%)	0.086
— Sustained VT or VF	5 (28%)	12 (32%)	0.76
LVEF (%)	46 [38-55]	46 [31-60]	0.99
— Low LVEF	11 (61%)	30 (60%)	1.00
Cardiac device implantation	13 (72%)	36 (72%)	1.00
Successful corticosteroid therapy	7 (39%)	14 (28%)	0.53

CAVB : complete atrioventricular block, VT : ventricular tachycardia, VF : ventricular fibrillation,
Low LVEF: the patients with LVEF < 50% in the echocardiography at their enrollment

* : $p < 0.05$

Supplemental table S9. Associations between the risk HLA allele ***HLA-C*03:04*** and clinical characteristics

	**With** *HLA-C*03:04* (n = 5)	**Without** *HLA-C*03:04* (n = 63)	P
**Clinical characteristics**			
Age of onset (years)	61 [54-68]	63 [56-71]	1.00
Gender (female)	3 (60%)	40 (63%)	1.00
**Clinical phenotypes**			
CAVB	2 (40%)	28 (44%)	1.00
VT or VF	1 (20%)	24 (38%)	0.64
— Sustained VT or VF	1 (20%)	16 (25%)	1.00
LVEF (%)	43 [29-72]	46 [33-59]	1.00
— Low LVEF	3 (60%)	38 (60%)	1.00
Cardiac device implantation	3 (60%)	46 (73%)	0.61
Successful corticosteroid therapy	1 (33%)	20 (38%)	1.00

CAVB : complete atrioventricular block, VT : ventricular tachycardia, VF : ventricular fibrillation,
Low LVEF: the patients with LVEF < 50% in the echocardiography at their enrollment

* : $p < 0.05$

Supplemental table S10. Associations between the risk HLA haplotype ***DQA1*01:03_DQB1*04:01*** and clinical characteristics

	**With** DQA1*01:03_DQB1*04:01 (n = 12)	**Without** DQA1*01:03_DQB1*04:01 (n = 56)	P
**Clinical characteristics**			
Age of onset (years)	67 [56-76]	62 [54-70]	0.44
Gender (female)	7 (58%)	36 (64%)	0.75
**Clinical phenotypes**			
CAVB	8 (67%)	22 (39%)	0.11
VT or VF	7 (58%)	18 (32%)	0.11
— Sustained VT or VF	5 (42%)	12 (21%)	0.16
LVEF (%)	41 [30-50]	47 [36-60]	0.25
— Low LVEF	9 (75%)	32 (57%)	0.34
Cardiac device implantation	8 (67%)	41 (73%)	0.73
Successful corticosteroid therapy	5 (50%)	17 (37%)	0.49

CAVB : complete atrioventricular block, VT : ventricular tachycardia, VF : ventricular fibrillation,
Low LVEF: the patients with LVEF < 50% in the echocardiography at their enrollment

* : $p < 0.05$

Supplemental table S11. Associations between the risk HLA haplotype ***DPA1*02:02_DPB1*09:01*** and clinical characteristics

	**With** DPA1*02:02_DPB1*09:01 (n = 12)	**Without** DPA1*02:02_DPB1*09:01 (n = 56)	P
**Clinical characteristics**			
Age of onset (years)	66 [62-72]	60 [53-70]	0.16
Gender (female)	9 (75%)	34 (61%)	0.51
**Clinical phenotypes**			
CAVB	5 (42%)	25 (45%)	1.00
VT or VF	5 (42%)	20 (36%)	0.75
— Sustained VT or VF	4 (33%)	13 (23%)	0.48
LVEF (%)	45 [39-56]	46 [32-61]	0.96
— Low LVEF	9 (75%)	32 (57%)	0.34
Cardiac device implantation	8 (67%)	41 (73%)	0.73
Successful corticosteroid therapy	4 (36%)	17 (38%)	1.00

CAVB : complete atrioventricular block, VT : ventricular tachycardia, VF : ventricular fibrillation,
Low LVEF: the patients with LVEF < 50% in the echocardiography at their enrollment

* : $p < 0.05$

Supplemental table S12. Genes of interest for calculating the binding affinity to HLA class II molecules

<Cardiomyopathy related genes>

ABCC9, ACTC1, ACTN2, ANKRD1, BAG3, CACNA1C, CRYAB, CSRP3, DES, DMD
DSC2, DSG2, DSP, EMD, EYA4, FHL1, FHL2, FKTN, FLNC, GLA
ILK, JPH2, JUP,LAMA4, LAMP2, LDB3, LMNA, MYBPC3, MYH6, MYH7
MYL2, MYL3, MYLK3, MYPN, NEXN, PKP2, PLN, PRKAG2, PSEN1, PSEN2
PTPN11, RAF1, RBM20, RIT1, RYR2, SCN5A, SGCB, SGCD, TCAP, TMEM43
TNNC1, TNNI3, TNNT2, TPM1, VCL

<Arrhythmia related genes>

ACADM, ACADVL, AKAP9, BTNL2, BVES, CACNA1D, CACNA1S, CACNB2, CALM1, CALM2
CALM3, CASQ2, CDH2, CTNNA3, CYTB, DMPK, GATA4, GATA6, GNAI2, GPD1L
HCN4, KCND3, KCNE1, KCNE3, KCNJ18, KCNJ2, MYOZ2, POPDC2, SCN10A, SCN1B
SCN2B, SCN3B, SCN4B, TANGO2, TECRL, TRDN, TTR

<Genes highly expressed in human heart>

ACTA1, ALDOA, ATP5B, BSG, CKM, CKMT2, COX6A2, COX7A1, COX8A, EEF1A2
EEF2, FABP3, HSPB1, HSPB7, LDHB, MYL9, NDUFA1, NDUFS5, NMRK2, PTGDS
SLC25A4, FTL, GAPDH, RPL10, RPLP1, RPS11, RPS12, RPS16

<Genes highly expressed in human lung, liver, and kidney>

A2M, ACTB, ACTG1, B2M, CD74, IFITM3, S100A9, SFTPA1, SFTPA2, SFTPB
SFTPC, TMSB10, TMSB4X, ZFP36, ALB, AMBP, APOA1, APOA2, APOC3, CRP
FGA, FGB, FGG, HP, ORM1, RBP4, SAA1, SERPINA1, CTSD, DEFB1
EEF1A1, EEF1G, ENO1, FTH1, GPX3, IGFBP7, RPL19, RPL3, RPL8, RPS27
S100A6, SPP1, UMOD

<Heart failure related genes>

AST, ALT, LDH, ACE2, ADRB1, CHRM2, NPPA, NPPB, SLC12A3
